# Intraretinal variation in disease severity in the *Oat*^*rhg*^ mouse model of gyrate atrophy

**DOI:** 10.1016/j.exer.2025.110382

**Published:** 2025-04-08

**Authors:** Robin J. Wilder, Andrea F. An, Brent A. Bell, Georgia Fossett, Alaina M. Wojciechowski, Ivan Shpylchak, Katherine E. Uyhazi

**Affiliations:** F.M. Kirby Center for Molecular Ophthalmology, Scheie Eye Institute, Department of Ophthalmology, University of Pennsylvania Perelman School of Medicine, Philadelphia, PA, 19104, USA

**Keywords:** Retina, Gyrate atrophy, OAT, Ornithine aminotransferase, Retinal pigment epithelium

## Abstract

Gyrate atrophy is an autosomal recessive retinal degeneration caused by pathogenic variants in the gene encoding ornithine aminotransferase (OAT), a mitochondrial enzyme required for ornithine degradation. Deficiency of OAT leads to hyperornithinemia and progressive chorioretinal atrophy that results in permanent vision loss. Strict dietary arginine restriction can slow the progression of the disease, but long-term adherence to the diet is challenging and not curative. Here, we characterize the retinal structure and function of the retarded hair growth (*Oat*^*rhg*^) mouse model of gyrate atrophy in order to identify appropriate outcome measures for future therapeutic approaches. Optical coherence tomography (OCT), histological sections, and retinal pigment epithelium (RPE) flat mounts of 12-month-old *Oat*^*rhg*^ mice revealed a well-defined patch of atrophy in the superonasal and occasionally inferior retina, characterized by RPE cell mounding, migration, and hypertrophy. The remainder of the retina was indistinguishable from age-matched wild type controls, and full-field electroretinograms (ERGs) were not significantly different between *Oat*^*rhg*^ and wild type mice. Therefore, unlike mice harboring the perinatal-lethal null mutation in OAT (*O*at^Δ^) which exhibit a loss of central photoreceptor cells and decreased ERG signal starting at 4 months, the *Oat*^*rhg*^ mouse exhibits a milder phenotype with intraretinal variation in disease severity that is reminiscent of the regional predilection observed in patients. These structural abnormalities are not sufficient to negatively impact retina-wide function but are accessible to monitoring by multimodal retinal imaging for testing of novel treatments.

## 1. Introduction

Gyrate atrophy (GA) is an autosomal recessive retinal degeneration that results in progressive, permanent vision loss. GA affects approximately 1 in 1,500,000 individuals, with the highest incidence in Finland (1 in 50,000) (Montioli et al., 2021). Patients typically present with myopia and nyctalopia in early childhood, followed by posterior subcapsular cataract formation and loss of peripheral vision in the 20s (Balfoort et al., 2021; Montioli et al., 2021; Simell and Takki, 1973; Takki and Milton, 1981). Ophthalmologic exams reveal well-demarcated scalloped patches of chorioretinal atrophy that begin in the midperipheral retina and progressively coalesce towards the macula, resulting in central vision loss (Takki, 1974).

GA is caused by biallelic variants in the gene encoding ornithine aminotransferase (OAT), a mitochondrial enzyme essential for ornithine degradation. Dietary arginine is metabolized into ornithine, and OAT catalyzes the reversible reaction that converts ornithine into glutamate-5-semialdehyde (GSA). GSA is then converted to pyrroline-5-carboxylate (P5C), a precursor of proline and glutamate (Ginguay et al., 2017). Without functional OAT, systemic ornithine accumulates 10–15 fold higher than normal levels and is associated with retinal pigment epithelial (RPE) dysfunction and a progressive chorioretinal degeneration (Montioli et al., 2021; Simell and Takki, 1973; Wang et al., 1995). There is no cure for GA, but dietary arginine restriction can slow progression of the disease (Kaiser-Kupfer et al., 1980, 2002; Valle et al., 1980; Wang et al., 2000). However, the low-protein diet required to sufficiently reduce ornithine levels in human patients is extremely difficult to maintain and retinal degeneration progresses despite achieving normal plasma ornithine concentration (Vannas-Sulonen et al., 1987), thus highlighting a need for alternative therapeutic approaches.

Despite identification of the causative gene and a thorough characterization of the clinical and biomolecular features of GA, the mechanisms resulting in chorioretinal degeneration are poorly understood – illustrating the need for a physiologically representative mouse model of the disease. There are currently two available mouse models of gyrate atrophy: a knockout mouse (*Oat*^*Δ*^) that was generated via the targeted disruption of the murine *Oat* gene with a neomycin resistance cassette (Wang et al., 1995) and a *retarded hair growth* (*Oat*^*rhg*^) mouse that harbors a spontaneous missense mutation in *Oat* (Bisaillon et al., 2014). The *Oat*^*Δ*^ mouse has a pronounced retinal degeneration by 7 months of age, but due to a paradoxical transient neonatal *hypo*-ornithemia, these mice require intraperitoneal arginine injections twice a day for the first two weeks of life in order to survive (Wang et al., 1996, 2000). Conversely, the *Oat*^*rhg*^ mouse has improved survival, delayed postnatal growth and hair development, and retinal degeneration that occurs by 12 months of age that mimics OAT deficiency in humans (Bisaillon et al., 2014; Boffa et al., 2023). In fact, the glycine substitution at residue 353 that results in decreased OAT activity in the *Oat*^*rhg*^ mouse has been reported in at least one Spanish family with gyrate atrophy (Brody et al., 1992). This variant results in plasma ornithine levels comparable to the *Oat*^*Δ*^ mouse and OAT-deficient human patients (Bisaillon et al., 2014), thus representing a valuable preclinical model of the disease. In this study, we characterized the retinal structure and function of the *Oat*^*rhg*^ mouse in order to understand the natural history of the disease and identify appropriate outcome measures for testing future therapeutic approaches.

## 2. Methods

*B6Ei;AKR-Oat*^*rhg*^/J (Strain #003544) and wild type (WT) C57BL/6J mice were acquired from The Jackson Laboratory. All animals were bred and maintained in the animal facilities of The University of Pennsylvania under a 12-h light/12-h dark cycle with free access to food and water. Experiments were conducted in accordance with federal and institutional regulations on IACUC protocol #806931 and with the ARVO Statement for the Use of Animals in Ophthalmic and Vision Research. Mice were aged between 12 and 14 months prior to all experimental analyses and both male and female mice were used in these studies. To measure ornithine, blood was collected from the vena cava during a terminal procedure using a 21-gauge needle and a TB-syringe coated with UltraPure 0.5 M EDTA, pH 8.0 (Invitrogen,15575–038). Plasma was isolated by centrifugation (1200×*g* for 10 min at 4° C) and stored at −80 °C until analysis. Quantitation of amino acids was performed by the Penn Metabolomics Core using liquid chromatography/mass spectrometry and the addition of isotopically-labeled internal standards to the plasma samples.

For retinal imaging, mice were anesthetized with intraperitoneal ketamine (95–100 mg/kg) and xylazine (10 mg/kg). Corneas were anesthetized with topical 0.5 % proparacaine hydrochloride (Akron, Lake Forest, IL, USA), and pupils were dilated with one drop of a 1:1 mixture of 1 % tropicamide and 2.5 % phenylephrine (Akron, Lake Forest, IL). Media opacities were minimized by using ocular eye shields (Bell et al., 2014) and artificial tears (Refresh, Allergan, Irvine, CA, USA). *In vivo* retinal imaging was performed using a Spectralis HRA confocal scanning laser ophthalmoscope (cSLO; Heidelberg Engineering, Franklin, MA). cSLO images were captured using short-wavelength fundus autofluorescence (SW-FAF) (486 nm raster scanned laser for excitation and 500–680 nm bandpass range for emission collection) and near-infrared fundus autofluorescence (NIR-FAF) (795 nm raster scanned laser for excitation and 800 nm longpass for emission collection). The images were obtained using the 102 Ultrawide Field (UWF) lens, providing a ~3.4 mm field of view of the mouse fundus. Twenty-five frames were acquired in high-speed mode (768 × 768 pixels) from each mouse retina, and real-time co-registration and averaging were performed using the Heidelberg Eye Explorer (HEYEX 1) software’s automatic real-time (ART) processing feature. Averaged images were auto-normalized for optimal contrast. An SD-OCT system (Bioptigen Model R2200 Envisu; Leica Microsystems, Buffalo Grove, IL, USA) was used to obtain orthogonal radial volume scans (1.4-mm; 1000 A/scans × 2 B-scans × 15 frames @0° and 90° orientation) and averaged B-scans (15 frames) of the retina from the central (optic nerve centrally positioned within the en-face image), temporal, nasal, superior, and inferior quadrants. After acquisition, images underwent coregistration and averaging using Bioptigen Envisu Software v2.1.

Mice were dark-adapted overnight prior to electroretinography testing. Animals were anesthetized and dilated as above and placed on a 37-degree stage. Custom-made clear plastic contact lens electrodes were placed over each eye, and a platinum wire loop reference electrode was placed in the animal’s mouth. Full-field ERGs were recorded using a custom-built Ganzfeld dome and a computer-based system (Espion E2; Diagnosys) in a climate-controlled, electrically isolated dark room under dim red illumination. Band-pass filter cutoff frequencies were 0.3 Hz and 300 Hz. Experiments were started with dark-adapted ERGs elicited with increasing intensities of blue (467 nm; 0.01, 2.1, 5.0, and 90 cd s/m^2^) followed by a bright white flash (1584 cd s/m2) and 10-Hz flicker stimuli. 10–20 responses were averaged for each of the intensity steps, and amplitudes and timings of the a- and b-wave amplitudes ERGs were measured conventionally.

For histological studies, eyes were enucleated with the third eyelid intact for orientation and fixed in a combination of 2 % glutaraldehyde +2 % paraformaldehyde (PFA) at 4 °C. Eyecups were dissected, dehydrated with increasing concentrations of ethanol (75 %, 95 %, 100 %), infiltrated overnight in embedding solution (JB4 Solution A; Polysciences, Inc., Warrington, PA), and embedded in plastic resin (JB4; Poly sciences, Inc). 3 μm thick sections were prepared using a Leica RM2615 Rotary Microtome (Wetzlar, Germany), stained with toluidine blue, then dehydrated in a series of ethanol (95 %, 100 %) and xylenes. Stained sections were imaged on the Aperio Scanscope (20x images) and retinal morphology was assessed using Aperio ImageScope software (Leica Microsystems). To assess RPE flat mounts, eyes were enucleated, fixed in 4 % PFA, and carefully trimmed to remove the optic nerve and excess extraocular tissue. Eyecups were prepared by removing the anterior segment while leaving a small portion of the labeled superior cornea to preserve orientation. The neural retina was carefully dissected to expose the RPE. Eyecups were blocked in 10 % normal horse serum in TBS-T for 1 h at room temperature and then incubated overnight at 4 °C in primary antibody anti-ZO-1 (Invitrogen, 40–2200) at 1:200 in 2 % normal horse serum in TBS-T. Samples were washed and then incubated in secondary antibody (Alexa Fluor^™^ 488 goat anti-rabbit IgG: Invitrogen, A-11034) and Hoechst (10 μg/mL) for 1 h at room temperature. Samples were then fixed with 4 % PFA, washed, oriented, and mounted on a slide using ProLong^™^ Gold Antifade Mountant (Invitrogen, P36930). Slides were imaged with an Axio Imager M2 microscope (Zeiss, Oberkochen, Germany) equipped with an automatic stage for tiling images, epifluorescence, and Zen 3.8 (blue edition; v.3.8.99.05000) imaging software. ZO-1-stained RPE flat mount images were analyzed by ImageJ segmentation and particle analysis. Images were converted from 16-bit to 8-bit and the intensity threshold was adjusted via the automatic application of a thresholding algorithm based upon optimizing the contrast between ZO-1 labeled cell membranes and unstained cytoplasm. The Watershed separation algorithm was then used to delineate precise cell borders and the Analyze Particles function was used to calculate cell areas. Minimum and maximum thresholds for quantified cell areas were set at discretion to avoid the inclusion of artifacts of the segmentation process in the data.

## 3. Results and Discussion

**Fig. 1.**
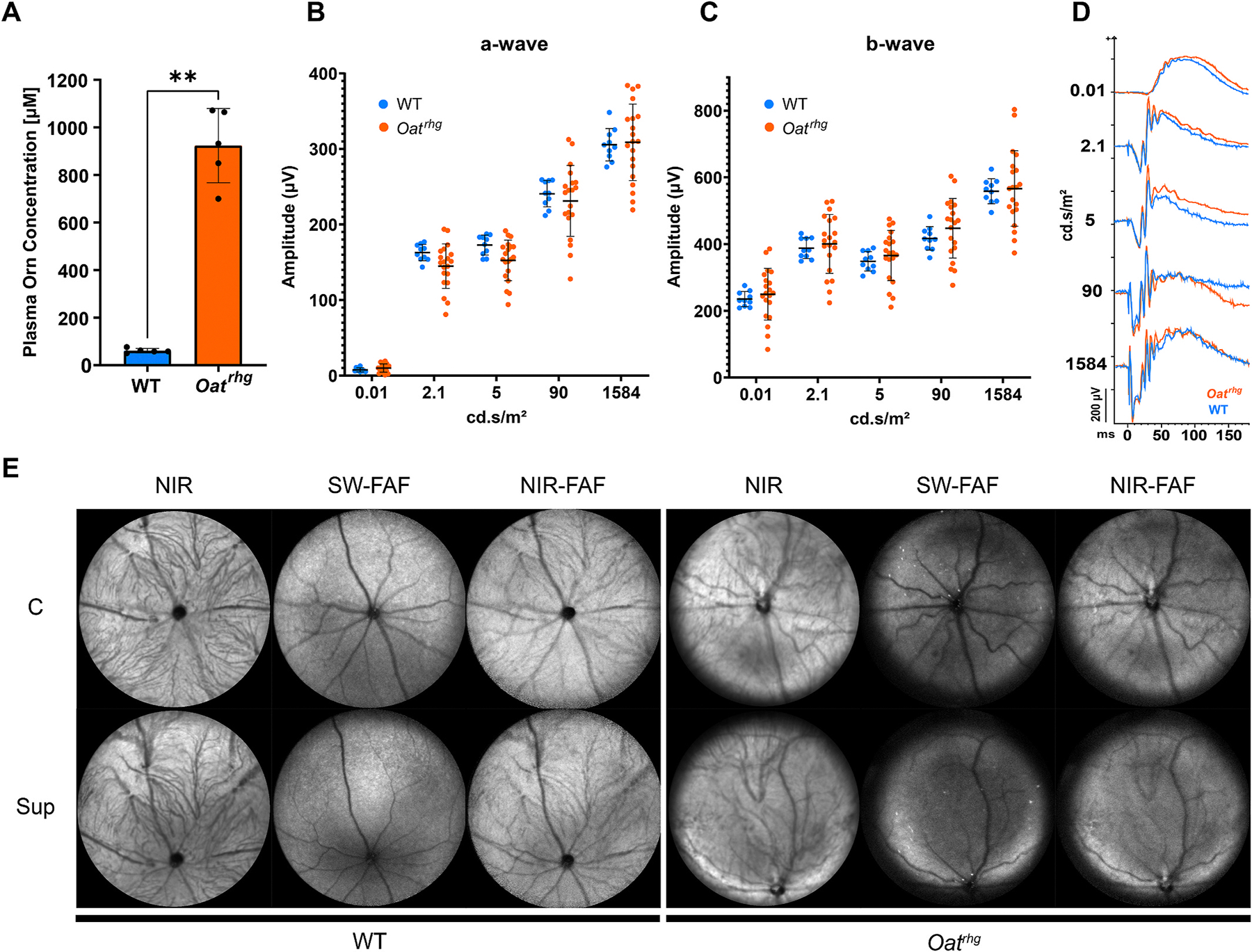
*Oat*^*rhg*^ mice have hyperornithinemia with minimal deficits in retina-wide structure and function. **(A)** Amino acid metabolic analysis indicates that *Oat*^*rhg*^ mice have plasma ornithine concentrations 15-fold higher than WT mice [p = 0.0079**; *OAT*^*rhg*^ (n = 5; avg: 923.16 μM), WT (n = 5; avg: 61.16 μM)]. **(B**–**D)** Full-field electroretinogram (ffERG) recordings show no significant difference in a-wave (**B**) and b-wave (**C**) amplitudes or timing (**D**) between 12- and 14-month-old *Oat*^*rhg*^ (n = 10 mice, 20 eyes, orange traces) and WT mice (n = 5 mice, 10 eyes, blue traces). **(E)** Representative confocal scanning laser ophthalmoscopy (cSLO) images of 12–14-month-old *Oat*^*rhg*^ mice are indistinguishable from age-matched WT mice. Central (C), superior (Sup), near-infrared (NIR), short-wavelength fundus autofluorescence (SW-FAF), near-infrared fundus autofluorescence (NIR-FAF). The two-tailed Mann Whitney test was used for statistical analysis. Error bars represent mean ± SD; ** indicates p ≤ 0.01. (For interpretation of the references to colour in this figure legend, the reader is referred to the Web version of this article.)

In this study we characterized retinal structure and function in the *Oat*^*rhg*^ mouse model of gyrate atrophy. *Oat*^*rhg*^ homozygous missense mutations were confirmed by PCR genotyping prior to all experiments. *Oat*^*rhg*^ mice had delayed growth and hair development, as well as a 15-fold increase in plasma ornithine concentration compared to WT mice [*Oat*^*rhg*^ (n = 5; avg: 923.16 μM), WT (n = 5; avg: 61.16 μM)] (Fig. 1A) as has been previously reported in this model (Bisaillon et al., 2014; Boffa et al., 2023). Interestingly, *Oat*^*rhg*^ mice also frequently demonstrated hyperactive and aggressive behavior, particularly evident in female mice. Although further behavioral characterization is beyond the scope of these studies, these findings are intriguing in the context of limited reports of neurocognitive impairment associated with gyrate atrophy (Valayannopoulos et al., 2009; Valtonen et al., 1999).

To investigate retinal function, dark-adapted full field electroretinogram (ffERG) recordings were performed on 12-month-old *Oat*^*rhg*^ and age-matched wild type mice. Although 2/10 *Oat*^*rhg*^ animals had decreased a- and b-wave amplitudes, ERG recordings demonstrated considerable inter-animal variability and there was no statistically significant difference in the average a-wave amplitude (Fig 1B), b-wave amplitude (Fig. 1C) or timing (Fig. 1D) between 12-and 14-month-old *Oat*^*rhg*^ mice (n = 20 eyes) and WT controls (n = 10 eyes). These findings differ from a recent report which showed a modest decrease in a- and b-wave ERG amplitudes in 12-month-old (but not 7-month-old) *Oat*^*rhg*^ mice (Boffa et al., 2023). This discrepancy may be due to natural variation in the retinal phenotype of aged *Oat*^*rhg*^ mice, similar to the variation found in patients with gyrate atrophy (Balfoort et al., 2024). Alternatively, these differences may be due to the mixed pigmented (C57BL/6JEi) and albino (AKR) background of *Oat*^*rhg*^ mice, in which the *rhg* mutation spontaneously arose in the AKR/J background and animals were subsequently outcrossed to C57BL/6JEi mice. Due to inherent changes in ERG signals in albino mice (Pinto et al., 2007), *Oat*^*rhg*^ mice with white fur have earlier impairment of b-wave amplitudes than their pigmented littermates. While only pigmented animals were used in both studies, ERG signals are influenced by variation in the background genetic composition of inbred mouse strains (Pinto et al., 2007), and it is possible that the mixed background of the *Oat*^*rhg*^ strain results in considerable variability in ERG waveforms. It is also possible that *Oat*^*rhg*^ mice aged beyond 14 months may have differences in ERG amplitudes compared to wild type mice. These findings highlight the importance of considering 1) background strain, 2) inter-animal variability, and 3) intraretinal variation in disease severity when choosing appropriate outcome measures and the number of replicates for future interventional studies.

**Fig. 2.**
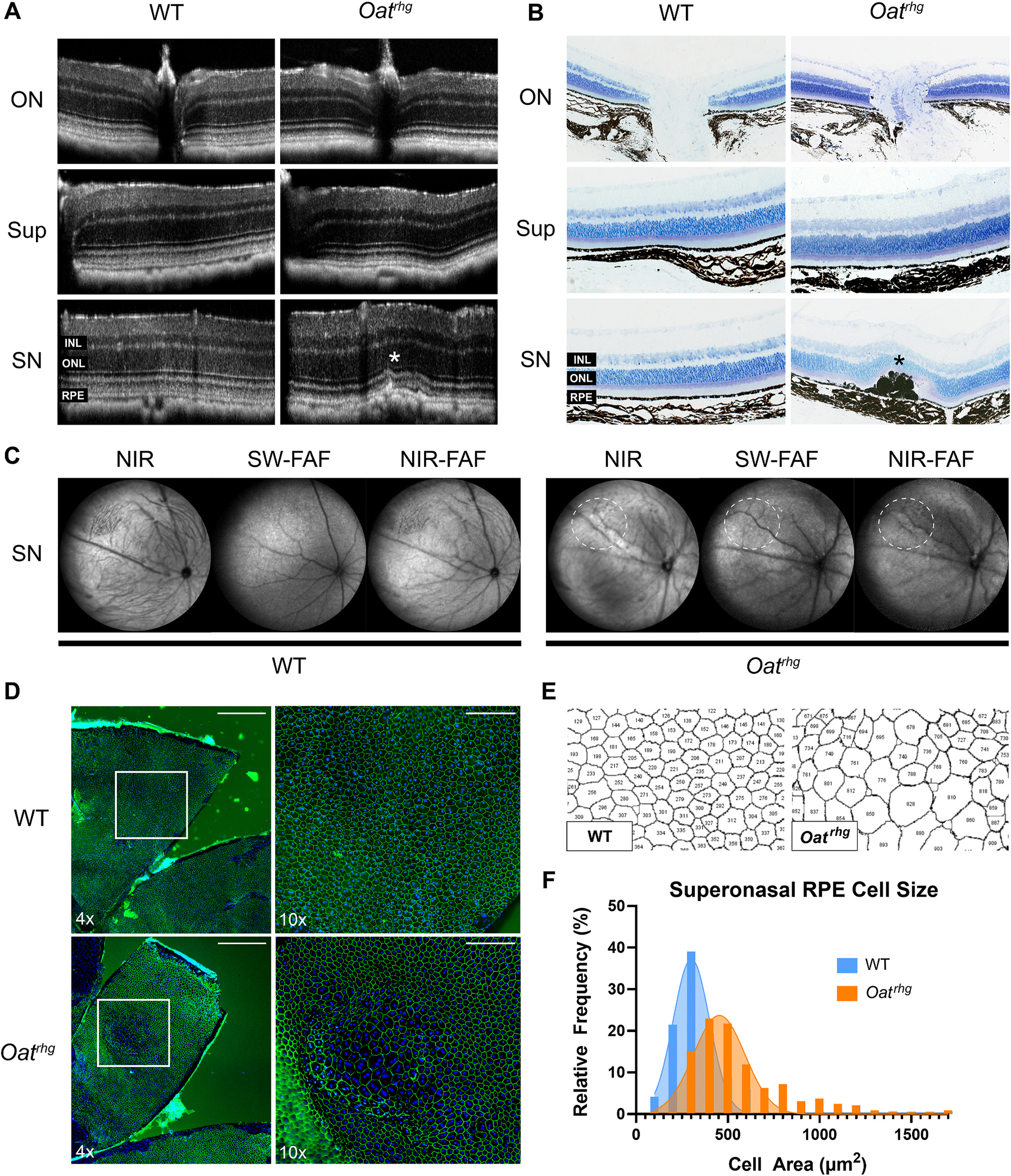
*Oat*^*rhg*^ mice have a distinct patch of atrophy in the superonasal retina. (**A-D**) Representative images of 12–14-month-old *Oat*^*rhg*^ and WT mice. **(A)** Optical coherence tomography (OCT) *in vivo* imaging reveals a focal disruption in outer retinal laminations in the superonasal (SN) retina of *Oat*^*rhg*^ mice (white asterisk) compared to age-matched WT controls. Central (ON) and superior (Sup) regions have normal retinal architecture. ONL: outer nuclear layer, INL: inner nuclear layer; RPE: retinal pigment epithelium. **(B)** Plastic sections demonstrate RPE cell mounding and migration (black asterisk) in the superonasal retina of *Oat*^*rhg*^ mice. **(C)** Representative cSLO images of 12–14=month-old *Oat*^*rhg*^ mice corresponding to regions of OCT abnormalities (white dashed circles) are indistinguishable from age-matched WT mice. **(D)** ZO-1 immunofluorescence staining of *Oat*^*rhg*^ RPE flat mounts reveals a hypertrophic patch of RPE cells in this region. Insets are shown at higher magnification; 4x scalebar = 500 μm; 10x scalebar = 100 μm. ZO-1 (green), Hoechst (blue). **(E)** Image J segmentation and particle analysis of ZO-1-stained RPE patch in *Oat*^*rhg*^ mice and a comparable retinal region of WT mice were used to quantify RPE cell area. Dissection artifacts were identified and excluded from quantification. **(F)** *Oat*^*rhg*^ mice have larger RPE cells (585.2 ± 273.3 μm^2^) in the superonasal retina compared to all other retinal quadrants (P < 0.0001, 338.2 ± 88.75 μm^2^) and WT mice (P < 0.0001, 330.6 ± 121.2 μm^2^). Cell area measurements were pooled from WT (n = 3) and *OAT*^*rhg*^ (n = 3) mice. The two-tailed Mann Whitney test was used for statistical analysis. Error bars represent mean ± SD. (For interpretation of the references to colour in this figure legend, the reader is referred to the Web version of this article.)

To further analyze the retinal structure in *Oat*^*rhg*^ mice, we performed confocal scanning laser ophthalmoscopy (cSLO) and optical coherence tomography (OCT) retinal imaging on 12–14-month-old *Oat*^*rhg*^ mice and age-matched wild type controls. To our knowledge this represents the first non-invasive *in vivo* imaging reported in an animal model of gyrate atrophy, which can be compared to similar imaging modalities in human patients. cSLO images were indistinguishable between *Oat*^*rhg*^ and age-matched WT mice (Fig. 1E). However, OCT imaging revealed a distinct patch of atrophy primarily in the superonasal retina (Fig. 2A, white asterisk). 13/18 *Oat*^*rhg*^ mice had an atrophic patch in the superonasal retina; 2 of these mice had an additional atrophic patch in the inferior retina, while 1/18 mice had detectable atrophy solely in the inferior retina. There were no corresponding changes on en-face imaging within these regions (Fig. 2C, white dashed circles). 4/18 mice did not have detectable atrophy by OCT, but given the relatively small size of the RPE changes it is likely that these regions were not completely captured by *in vivo* imaging.

These changes were further investigated by histological plastic sections, which revealed disorganization of the outer retinal laminations with RPE mounding, migration, and focal thinning of the overlying outer nuclear layer (Fig. 2B, black asterisk). Prior studies reported subtle RPE abnormalities in 7-month-old *Oat*^*rhg*^ RPE mice (Bisaillon et al., 2014), suggesting that these patches develop between 7 and 12 months of age. Immunofluorescent staining of *Oat*^*rhg*^ RPE flat mounts for Zonula occludens-1 (ZO-1), a major structural component of tight junctions in RPE cells, was used to characterize RPE cell morphology (Fig. 2D). ZO-1 staining revealed a patch of hypertrophic RPE cells in the superonasal retina of *Oat*^*rhg*^ mice with an average length of 584.3 ± 44.5 μm (Fig. 2D, lower panel). RPE hypertrophy was also rarely seen in the inferior retina of *Oat*^*rhg*^ mice, but not in any other retinal quadrants or in wild type mice, which demonstrated characteristic uniform hexagonal RPE cell morphology (Fig. 2D, upper panel). ImageJ segmentation and particle analysis (Fig. 2E) was used to quantify the RPE cell area of the ZO-1-stained RPE flat mounts, which was significantly larger within the superonasal patch (585.2 ± 273.3 μm^2^) compared to all other retinal quadrants (338.2 ± 88.75 μm^2^, p < 0.0001) and RPE of WT mice (330.6 ± 121.2 μm^2^, p < 0.0001) (Fig. 2F).

Taken together, these findings suggest that the superonasal retina is particularly vulnerable to the absence of ornithine aminotransferase in the *Oat*^*rhg*^ model of gyrate atrophy. Importantly, our data suggest that the RPE swelling and doming reported previously in this model does not occur uniformly throughout the retina, but rather occurs in a small superonasal (and sometimes inferior) patch. These focal findings are consistent with other models of RPE metabolic dysfunction (Chen et al., 2016; Zhao et al., 2011) and chemically-induced retinal injury (Anderson et al., 2024) and suggest that RPE cell death is occurring in these regions, resulting in phagocytosis by retinal macrophages and the hypertrophy of surrounding cells to maintain the RPE monolayer. It is unclear why the superonasal retina is most affected; there are no known differences in OAT expression throughout different anatomical regions of the retina, but such intraretinal variability in disease is common in inherited retinal degenerations and may be due to regional differences in chorioretinal blood supply, metabolic demand, RPE cell subpopulations, or light exposure (Brown et al., 2019; Napoli et al., 2021; Ortolan et al., 2022).

There was mild thinning of the outer nuclear layer (ONL) directly overlying regions of abnormal RPE cells, but we did not observe wide-spread shortening of photoreceptor outer segments or loss of ONL thickness in the *Oat*^*rhg*^ mouse, as has been reported in the *Oat*^Δ^ mouse and the human disease. Since RPE dysfunction likely precedes photoreceptor loss and secondary atrophy of the choriocapillaris, the phenotype of the *Oat*^*rhg*^ mouse is consistent with an earlier stage of disease, at a time when RPE abnormalities have not yet led to photoreceptor cell death. In fact, RPE cells showed the earliest histopathological changes in the *Oat*^Δ^ mouse (Wang et al., 1996), which has a more severe phenotype due to the null allele compared to the missense variant present in the *Oat*^*rhg*^ mouse. Similarly, ERG recordings in human patients with gyrate atrophy have shown detectable a- and b-waves with loss of the c-wave (Takki, 1974), suggesting that RPE dysfunction occurs before photoreceptor dysfunction. Post-mortem histological findings in a patient with gyrate atrophy also revealed regions of intact photoreceptors overlying abnormal RPE (Wilson et al., 1991). In light of these observations, it is possible that *Oat*^*rhg*^ mice aged beyond 12–14 months or on a different genetic background may eventually manifest subsequent outer retinal changes.

Furthermore, we have shown that the majority of the retina has a normal structure as assessed by OCT and histology, and that ERG a-wave and b-wave amplitudes from 12- to 14-month-old *Oat*^*rhg*^ mice are highly variable but indistinguishable from wild type mice. Since full-field ERG testing represents an average signal across the entire retina, this technique cannot distinguish small patches of abnormal retinal function and thus may not be an ideal outcome measure for functional testing in this particular model. Since the retinal atrophy was detectable by OCT, non-invasive retinal imaging may prove useful for future studies and could be extrapolated to OCTs from patients with gyrate atrophy.

## Data Availability

Data will be made available on request.
